# Roles of mitochondria in neutrophils

**DOI:** 10.3389/fimmu.2022.934444

**Published:** 2022-08-19

**Authors:** Ziming Cao, Meng Zhao, Hao Sun, Liang Hu, Yunfeng Chen, Zhichao Fan

**Affiliations:** ^1^ Department of Immunology, School of Medicine, UConn Health, Farmington, CT, United States; ^2^ Arthritis and Clinical Immunology Program, Oklahoma Medical Research Foundation, Oklahoma City, OK, United States; ^3^ Department of Microbiology and Immunology, University of Oklahoma Health Science Center, Oklahoma City, OK, United States; ^4^ Department of Medicine, University of California San Diego, La Jolla, CA, United States; ^5^ Academy of Integrative Medicine, Shanghai University of Traditional Chinese Medicine, Shanghai, China; ^6^ Department of Biochemistry and Molecular Biology and Department of Pathology, University of Texas Medical Branch, Galveston, TX, United States

**Keywords:** neutrophils, mitochondria, respiratory burst, NETosis, migration, adhesion

## Abstract

Neutrophils are the most abundant leukocyte in human blood. They are critical for fighting infections and are involved in inflammatory diseases. Mitochondria are indispensable for eukaryotic cells, as they control the biochemical processes of respiration and energy production. Mitochondria in neutrophils have been underestimated since glycolysis is a major metabolic pathway for fuel production in neutrophils. However, several studies have shown that mitochondria are greatly involved in multiple neutrophil functions as well as neutrophil-related diseases. In this review, we focus on how mitochondrial components, metabolism, and related genes regulate neutrophil functions and relevant diseases.

## Introduction

Neutrophils play a central role in the innate immune system. They are the most abundant circulating white blood cells in human blood. They are recruited to infection sites or damaged tissue by a recruitment cascade that involves rolling, arrest, spreading, intravascular crawling, transendothelial migration, and in-tissue migration/chemotaxis (1–4). Neutrophils have various methods to kill invading microorganisms, including phagocytosis (5–7), neutrophil extracellular traps (NETs; and process of NETosis) (6, 8, 9), degranulation (6, 10), and respiratory burst (11, 12).

**Figure 1:**
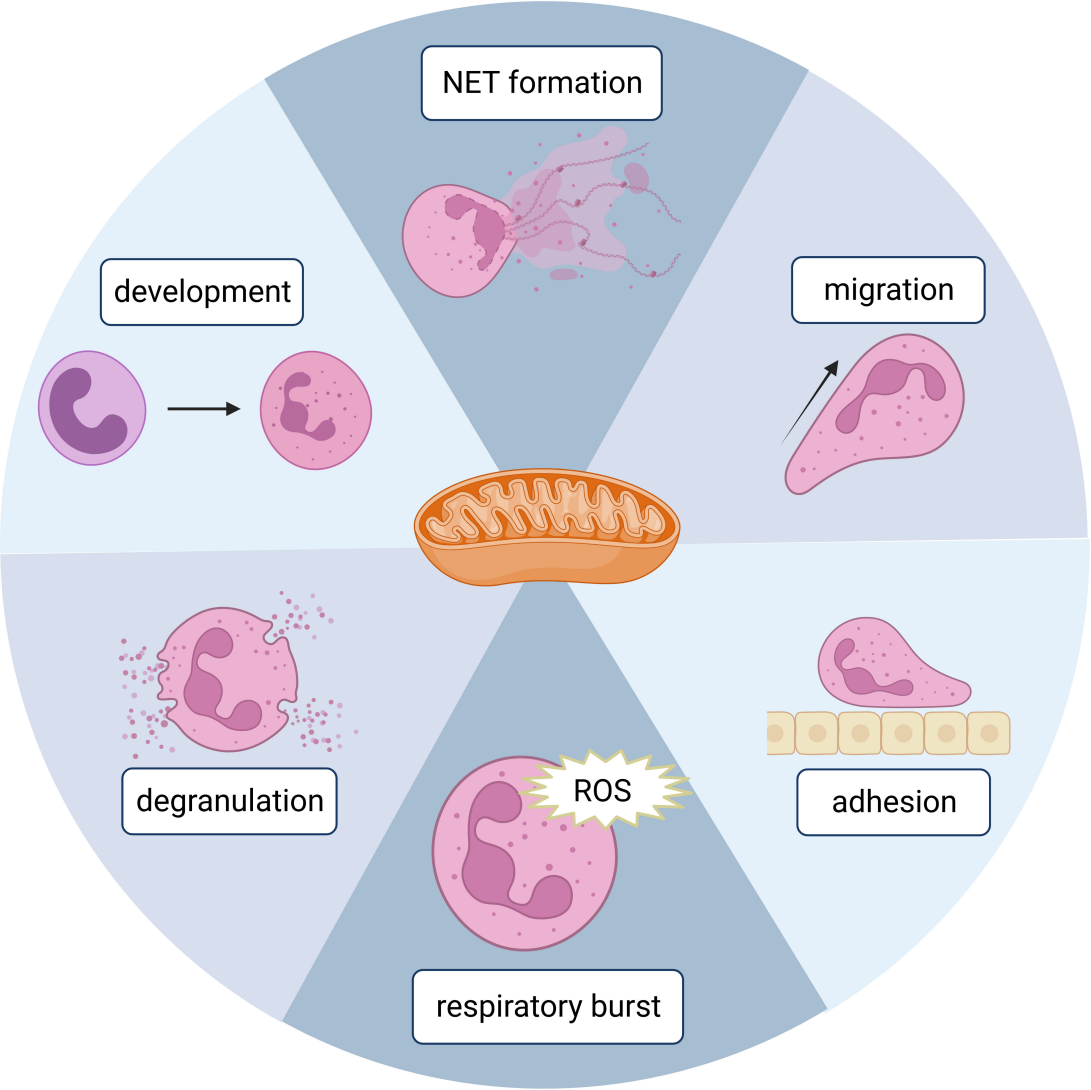
A schematic figure showing the involvement of mitochondria in different neutrophil functions. Created in BioRender.com.

Mitochondria are one of the most important organelles in eukaryotic cells. They are the powerhouses of eukaryotic cells since this is where adenosine triphosphate (ATP) is produced efficiently by oxidative phosphorylation (OXPHOS). Mitochondria are also central signaling hubs that regulate cell death (13). Until 2003, mitochondria had been underestimated for their role in neutrophils. Utilizing multiple specific dyes, Fossati et al. identified mitochondria in neutrophils as complex networks extending through the cytoplasm (14), which kickstarted intensive research on neutrophil mitochondria. Mitochondria are now known to have several important functions in neutrophils, such as NET formation, adhesion/migration, respiration burst, development/differentiation, and death, which will be discussed in this review (**Figure 1**). We will also discuss diseases related to dysfunctions of neutrophil mitochondria.

## Respiratory burst

Neutrophil respiratory burst, which involves the production of reactive oxygen species (ROS), plays an important role in controlling infections and the progression of inflammatory diseases. Here, the term ROS refers to reactive non-radical derivatives of molecular oxygen, such as H_2_O_2_, and reactive free radical species, such as O_2_^–^.

N-Formyl peptides (e.g., N-formyl-L-methionyl-L-leucyl-phenylalanine; fMLP) can stimulate neutrophil oxidative burst and produce ROS *via* formyl peptide receptors (FPRs) (15). Oligomycin and cyanide m-chlorophenylhydrazone (CCCP) that inhibit mitochondrial OXPHOS can reduce fMLP-induced neutrophil ROS production (15). Dihydrorhodamine (DHR) 123 was used to measure ROS production in this study. However, since mitochondrial OXPHOS also produces ROS (mitochondrial ROS, mROS), whether DHR123-indicated ROS are from mitochondrial OXPHOS or oxidative burst is unclear (15).

Another study showed that scavenging of mROS with mitochondria-targeted antioxidant SkQ1 could decrease neutrophil intracellular ROS upon cytochalasin D/fMLP stimulation in a dose-dependent manner (16). Then they investigated whether mROS directly contributes to the intracellular ROS or regulates intracellular ROS through an indirect mechanism. Intracellular ROS was labeled by the redox-sensitive dye 2′,7′-dichlorofluorescein-diacetate (DCFH-DA), which can be cleaved by intracellular esterases to form dichlorofluorescein and become fluorescent upon oxidation. Using membrane-impermeable catalase to scavenge extracellular ROS, they demonstrated that fMLP predominantly induced extracellular ROS formation, and the DCFH-DA-labeled intracellular ROS was coming from the penetration of extracellular ROS but not mROS. They also used MitoSOX to specifically label mROS, which were shown not to significantly contribute to fMLP-induced intracellular ROS accumulation. Thus, these results indicated that mROS does not directly contribute to intracellular ROS but is implicated in the NADPH oxidase (NOX)-dependent generation of extracellular ROS or transportation of extracellular ROS to the cytoplasm (16). Other mechanisms of how mROS regulate oxidative burst need further investigation. This study also found that SkQ1 can inhibit cytochalasin D/fMLP-induced degranulation based on decreased surface expression of azurophilic granule marker CD63 and specific granule marker CD66b, suggesting that mROS may influence granule exocytosis (16).

A recent study found that inhibition of complex III (cytochrome c reductase) with antimycin A can significantly reduce neutrophil superoxide O_2_^–^ production (17). MitoTEMPO can accumulate in mitochondria and reduce superoxide. Pre-incubation of neutrophils with MitoTEMPO can significantly attenuate neutrophils’ ability to kill *Staphylococcus aureus.* This effect is not enhanced by co-incubation with MitoTEMPO and antimycin A, suggesting that electron transport complex III is critical for mROS production and required for the bactericidal function of neutrophils (17). Rice et al. found that mitochondria contribute to prolonged H_2_O_2_ production. Moreover, if neutrophils have limited glucose, mitochondria can utilize fatty acid metabolism to maintain NADPH levels to support NOX-dependent respiratory burst instead of producing mROS (18).

## NET formation

NETs were discovered while studying the unique antibacterial mechanisms of neutrophils. When neutrophils were stimulated with interleukin-8 (IL-8), phorbol 12-myristate 13-acetate (PMA), or lipopolysaccharide (LPS), Brinkmann et al. found that extracellular filamentous structures containing DNA and histones can ensnare bacteria, such as *Staphylococcus aureus*, *Salmonella typhimurium*, or *Shigella flexneri* (8). The process of formation of NETs is called NETosis, which is a unique form of cell death in neutrophils (19). NETosis is essential for the host to fight against microorganisms. However, it also contributes to many pathological processes, including cancer, thrombosis, and autoimmune diseases (20, 21).

**Figure 2:**
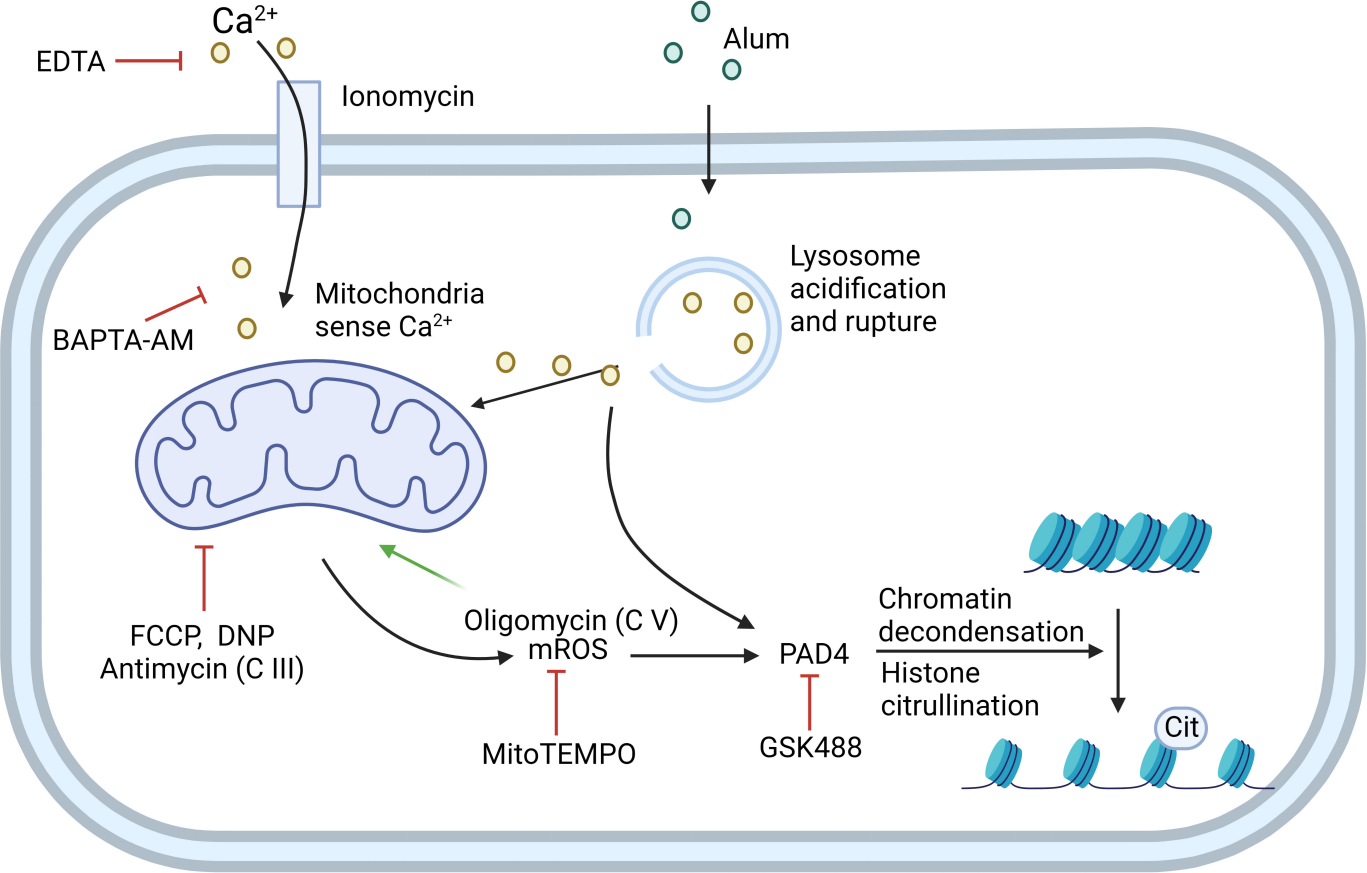
NOX-independent NETosis can be induced by ionomycin or alum. Ionomycin works as a Ca^2+^ ionophore to transfer extracellular calcium (Ca^2+^) into cells. Alum causes lysosome acidification and rupture, which then leads to Ca^2+^ release. Mitochondria sense the elevation of Ca^2+^ in the cytoplasm and generate mitochondrial ROS (mROS). Cytoplasmic Ca^2+^ and mROS are both required to activate PAD4, which further induces histone citrullination and chromatin decondensation. EDTA and BAPTA-AM can chelate extracellular and cytoplasmic Ca^2+^, respectively, to inhibit this process. Mitochondrial inhibitor FCCP, DNP, and mitochondria complex III inhibitor antimycin can inhibit this process. mROS-specific scavenger MitoTEMPO also inhibits the pathway. Mitochondria complex V inhibitor oligomycin inhibits the retrograde proton transport, therefore leading to the increased MMP which drives enhanced NETosis. GSK488 is an inhibitor of PAD4. C III: complex III; C V: complex V. Created in BioRender.com.

Multiple stimuli can initiate NET formation, including nicotine (22), LPS, granulocyte-macrophage colony-stimulating factor (GM-CSF), PMA, ionomycin, and alum. However, each initiates NET formation using a different mechanism, which can be categorized into two types based on the dependence of NOX: NOX-dependent and NOX-independent (**Figure 2**).

PMA has been used as a stable NETosis inducer to study the mechanisms of NOX-dependent NETosis. The indispensable role of NOX was shown by using the NOX2 inhibitor DPI to significantly reduce PMA-induced NETosis, and that neutrophils from chronic granulomatous disease (CGD) patients—that have mutations in one of the genes encoding the components of the Nox2 NADPH oxidase complex—lose their ability for NETosis (19). PMA activates protein kinase C (PKC), which subsequently activates NOX2 through c-Raf/MEK/Erk to produce cytosolic ROS (23). The release of neutrophil elastase (NE) and myeloperoxidase (MPO) follows the production of ROS, causing specific histone degradation and chromatin decondensation. However, signaling components between ROS and translocation of NE from azurophilic granules and to the nucleus are not clear (24). During this process, although PMA can induce the production of mROS, PMA-induced mROS cannot drive NETosis (25). Why ROS from different sources have distinct effects on NETosis is not clear.

PMA-induced NOX-dependent NETosis needs glucose. Human neutrophils cultured in glucose-free media are unable to form NETs. Further, glycolysis inhibitor 2-deoxyglucose, but not OXPHOS inhibitor oligomycin, abolishes neutrophil’s ability to undergo PMA-induced NETosis (26). GM-CSF plus complement factor 5a (C5a)/LPS-dependent NET formation is also NOX-dependent, which is demonstrated by using NOX inhibitor DPI and neutrophils from CGD patients (27). However, it has also been shown that electron transport complex I localized on the inner membrane of mitochondria (IMM) is critical for GM-CSF plus C5a/LPS-dependent NET formation (28). This is because complex I produces nicotinamide adenine dinucleotide (NAD+), which participates in glycolysis to generate ATP. Blocking complex I with rotenone or piericidin A but not complex III or complex V leads to the inability of mouse and human neutrophils to release DNA upon stimulation with GM-CSF plus C5a (28). Optic atrophy 1 mitochondrial dynamin-like GTPase (OPA1) is one of the “mitochondria-shaping” proteins, bound to the outer space of the inner mitochondrial membrane. Patients with mutated OPA1 can develop autosomal dominant optic atrophy (ADOA). The neutrophils from some ADOA patients or OPA1-deficient mice cannot release DNA following GM-CSF plus C5a stimulation. Amini et al. further found that deficiency of OPA1 leads to the reduced activity of complex I, therefore reducing ATP production by limiting NAD+ availability in glycolysis, resulting in the inability to release DNA to extracellular space and failure of the mitochondrial network formation (28).

Calcium ionophore ionomycin and A23187 can drive NETosis *via* a NOX-independent pathway. mROS, but not NOX2-dependent ROS, is required for NOX-independent NETosis in dHL60 cells and human neutrophils (29). Mitochondrial uncouplers, such as 2,4-dinitrophenol (DNP) and carbonylcyanide-p-trifluoromethoxyphenylhydrazone (FCCP), dissipate the proton gradient and inhibit mitochondrial ATP synthesis. DNP and FCCP inhibit NOX-independent NETosis in a dose-dependent manner (29). Takishita et al. used dideoxycytidine to generate mitochondria-deficient *ρ* ^0^ cells from HL60 cells. They also found that *ρ* ^0^ cells release less DNA compared to neutrophil-like HL60 cells after stimulating with A23187, but not PMA, suggesting that mitochondria are crucial for NOX-independent NETosis (30). MitoTEMPO can attenuate A23187-induced NET formation, suggesting that mROS is the key part of the NOX-independent pathway (30, 31).

Reithofer et al. proposed a mechanism of alum-induced NETosis regarding mitochondrial membrane potential as the major driving force (32). They first found that positively charged alum has a similar effect with calcium ionophore ionomycin on activating NETosis. However, alum-induced NETosis does not require extracellular Ca^2+^ like ionophores. Instead, the rupture of lysosomes followed by the release of lysosome-stored Ca^2+^ leads to an increased production of mROS. The authors further investigated the role of the respiration chain in mROS production and NETosis. They found that rotenone, the inhibitor of complex I, does not affect alum-induced NETosis, whereas antimycin, an inhibitor of complex III, decreased alum-induced NETosis to a similar extent as DNP. The inhibition of complex V inhibits retrograde proton transport, increases mitochondrial membrane potential, and enhances alum-induced NETosis. This suggests that alum/ionophore-induced NETosis uses a different pathway from C5a/LPS-induced NETosis, which is dependent on complex I-controlled, glycolytic ATP production (32).

Ca^2+^ signals were found important for NOX-independent NETosis. Mitochondria will sense Ca^2+^ either from extracellular space or intracellular components like ER or lysosome and produce ROS in response to that. The uptake of Ca^2+^ by the mitochondrial calcium uniporter (MCU) is regulated by mitochondrial calcium uptake 1 (MICU1), which acts as a gatekeeper for Ca^2+^ flux through the MCU. In the MICU1 conditional KO model, MCU-mediated ion flux coincides with increased production of mitochondrial O_2_^-^ in response to *S. aureus*, thereby causing MICU1 knockout neutrophils to undergo accelerated and more robust suicidal NETosis and decreased azurophilic granule degranulation (33). Both pharmacological and genetic evidence showed that peptidylarginine deiminase 4 (PAD4) is critical for chromatin decondensation and NETosis (34, 35). Activation of PAD4 requires both ROS and Ca^2+^ signaling (21).

Although NETs are mainly composed of chromosomal DNA, mitochondrial DNA (mtDNA) can be released to extracellular space stimulated by GM-CSF C5a (27) or ribonucleoprotein immune complexes (36), suggesting a direct involvement of mitochondria in NET formation.

Current methods used to dissect the NETosis pathway are mainly pharmacological inhibition due to the short life of neutrophils. Here, we summarize studies showing cellular components participating in the pathway in **Table 1**.

**Table 1:** NETosis regulators.

Cell type	Stimuli	Component	Inhibitor	References
Mouse peripheral neutrophil	Ionomycin (NOX-independent)	PAD4	GSK488	(34)
Human peripheral neutrophil	S*. aureus*	PAD4	GSK488	(34)
Mouse peripheral neutrophil	LPS, H_2_O_2_, PMA	PAD4	knockout	(35)
Human peripheral neutrophil	PMA	NOX2	DPI	(29)
	A23187 (NOX-independent)	mROS	DNP	(29)
		mitochondria	FCCP	(29)
dHL 60	PMA	ERK	FR180204	(29)
		Akt	XI	(29)
	A23187 (NOX-independent)	Akt	XI	(29)
		mROS	DNP, FCCP	(29)
		SK3 channel	siRNA	(29)
Human peripheral neutrophil	A23187, ionomycin (NOX-independent)	mROS	MitoTEMPO	(31)
Human peripheral neutrophil	Nicotine (NOX-independent)	Akt	XI	(22)
		PAD4	CI-amidine	(22)
Mouse peripheral neutrophil	PMA	NOX2	Gp91phox knockout	(22)
Human peripheral neutrophil		c-Raf	GW5074	(23)
		MEK	U0216	(23)
		ERK2	Peptide inhibitor	(23)
		PKC	Staurosporine	(23)
Human peripheral neutrophil	Ionomycin (NOX-independent)	Extracellular Ca^2+^	Ca^2+^-free PBS	(32)
	Alum (NOX-independent)	Intracellular/lysosomal Ca2+	BAPTA-AM	(32)
		Lysosomal Ca^2+^	Bafilomycin	(32)
		mROS	DNP	(32)
		PAD4	GSK484	(32)
		Mitochondrial complex III	Antimycin	(32)
Human peripheral neutrophils	IL-18 (NOX-independent)	mROS	MitoTEMPO	(37)
		Ca2+	BAPTA-AM	(37)

## Migration and adhesion

**Figure 3:**
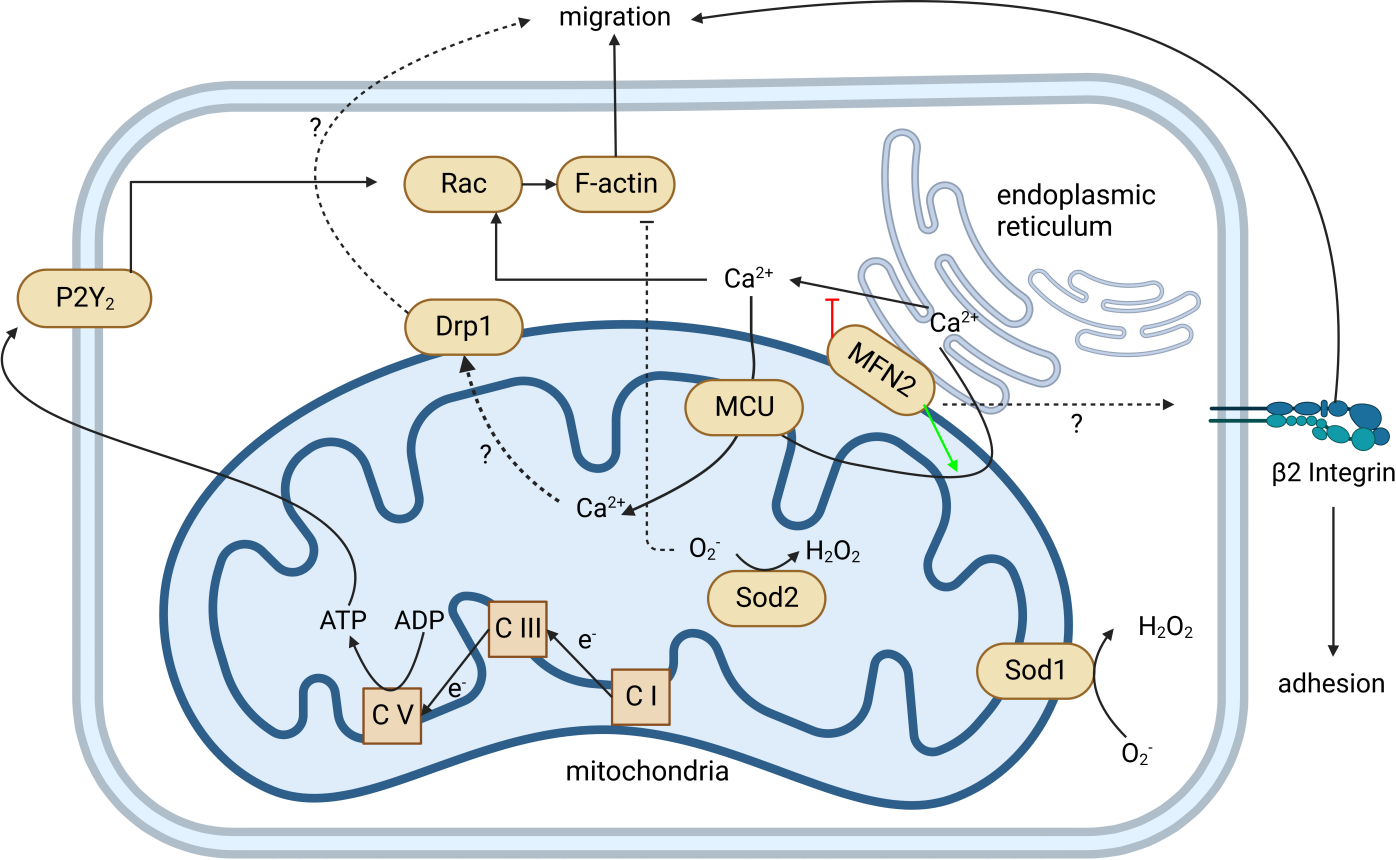
Mitochondria regulate neutrophil chemotaxis. Neutrophil mitochondria produce ATP to fuel neutrophil autocrine purinergic signaling, which is required for neutrophil chemotaxis. The production of ATP by mitochondria is through OXPHOS, coupled with an electron transport chain from complex I (C I) to complex V (C V). This is demonstrated by complexes I, III (C III), and V-specific inhibitors and/or gene knockouts. The P2Y2 receptor can recognize ATP, activate Rac, induce actin polymerization, and guide neutrophil migration. MFN2 can regulate *β*2 integrin activation through an unknown mechanism that affects both neutrophil adhesion and migration. MFN2 also tethers the endoplasmic reticulum (ER) with mitochondria and promotes ER Ca^2+^ transfer into mitochondria. Loss of MFN2 may lead to more ER Ca^2+^ released into the cytoplasm. Elevated cytoplasmic Ca^2+^ hyperactivates Rac and impairs the directionality of neutrophil migration. MCU mediates cytoplasmic and ER Ca^2+^ transferring into mitochondria and regulates downstream Drp1 through an unclear mechanism. Both MCU and Drp1 are involved in neutrophil migration. Sod1 and Sod2 mediate the reduction of O_2_^-^ to H_2_O_2_. Loss of Sod1 or Sod2 impairs the chemotaxis of neutrophils. Created in BioRender.com.

As the first-line defenders, neutrophils are critical for the innate immune system. The premise of nearly all functions of neutrophils is their ability to navigate and migrate to the infection or wound site. Mitochondria are one of the most important organelles in eukaryotic cells and work as a central hub for signal transduction and metabolism in the locomotion of most cells. Although mature neutrophils mainly rely on glycolysis for ATP (38), mitochondria still play an essential role in neutrophil locomotion (**Figure 3**). Inhibition of mitochondria by CCCP significantly reduced the migration speed of neutrophils and abolished chemotaxis (39). During chemotaxis, neutrophils release ATP from the leading edge of their cell surface to amplify chemotactic signals and direct cell orientation by feedback through P2Y2 nucleotide receptors and purinergic signaling (40). Mitochondria translocate to the front of the neutrophil, producing ATP to fuel the purinergic signaling (39). Meanwhile, P2Y2 receptors promote mTOR signaling and augment mitochondrial activity near the front of the cell (39). However, excessive mitochondrial ATP production induced by LPS impairs neutrophil chemotaxis (41). Unlike fMLP gradient-induced neutrophil ATP release from the leading edge of the cell surface, LPS uniformly disorganized intracellular trafficking of mitochondria, resulting in global ATP release that disrupted purinergic signaling, cell polarization, and chemotaxis. LPS-primed neutrophils cannot translocate mitochondria toward the leading edge in response to the fMLP gradient, but they can move their mitochondria uniformly toward the cell periphery. Neutrophil chemotaxis is impaired due to the lack of polarity. Removal of excessive extracellular ATP by adding apyrase can restore the chemotaxis of LPS-primed neutrophils (41). This study may partially explain the decreased chemotactic activity in neutrophils from septic patients compared to healthy controls (42). Zhou et al. first provided *in vivo* evidence that mitochondria regulate neutrophil motility in a zebrafish model (43). The gene encoding mtDNA polymerase, *polg*, was knocked out in a neutrophil-specific transgenic line and reduced the migration speed of neutrophils (43).

Intracellular NOX2-dependent ROS regulates neutrophil migration by modulating actin dynamics *via* inducing actin glutathionylation (44). Rotenone and metformin, the mitochondria complex I inhibitors, impair the electron transport chain, increase intracellular superoxide and hydrogen peroxide, and significantly reduce neutrophil recruitment in an acute LPS-induced lung inflammation mouse model (43, 45). Both mitochondria complex I inhibitor rotenone and complex III inhibitor antimycin significantly reduced neutrophil motility in zebrafish (43). Gene knockouts of *ndufs2* and *uqcrc1*, which are core components in complex I and III, respectively (46, 47), also resulted in a neutrophil motility reduction (43). Mitochondrial superoxide dismutase 1 and 2 (Sod1 and Sod2) mediate ROS reduction. The loss of Sod1 or 2 significantly reduces the speed of neutrophil migration, which can be rescued by N-acetylcysteine and/or MitoTEMPO. This implies that an excessive amount of intracellular ROS can inhibit neutrophil migration (43). However, this is in contrast to a certain condition where increased intracellular ROS may associate with increased neutrophil migration. Hv1/VSOP is a voltage-gated proton channel that mediates the extrusion of protons and supports intracellular ROS production (48). As expected, Hvcn1-deficient neutrophils produce more intracellular ROS. However, they exhibit stronger chemotaxis toward a low (0.25–1 µM) but not higher (10–50 µM) concentration of fMLP compared with wild-type (WT) neutrophils. Hvcn1-deficient neutrophils have a similar fMLP receptor expression and similar intracellular Ca^2+^ flux after fMLP stimulation compared to WT neutrophils. However, increased ERK activation was observed in Hvcn1-deficient neutrophils. The migration increase and ERK activation of Hvcn1-deficient neutrophils are NOX-dependent, supported by NADPH oxidase inhibitor DPI (49). Interestingly, they did not observe a change in migration of WT neutrophils treated with DPI, suggesting that the above migration changes in mitochondrial defect neutrophils might not be ROS-dependent. However, DPI has off-target effects where it inhibits cell redox metabolism and induces oxidative stress (50).

Recent work has highlighted the role of mitochondrial calcium homeostasis in regulating cell migration (51). Mitochondrial calcium uniporter (MCU), located on IMM, is responsible for mitochondrial calcium uptake. MCU knockdown impaired the migration of Hs578t cells, similar to the outcome of treatment with Ru360, a potent MCU inhibitor. This finding suggests that mitochondrial Ca^2+^ intake regulates cell migration. However, the study of MCU in neutrophils is limited by pharmacological treatment. Two independent groups have shown that MCU regulates neutrophil migration. Activation of MCU by treatment of spermine can promote primary human neutrophil chemotaxis; inhibition of MCU by Ru360 impairs the chemotaxis of human neutrophils (52) and DMSO-differentiated HL60 (dHL60) cells (43). Dynamin-related protein (Drp1) regulates mitochondrial fission, maintaining mitochondria morphology. In some circumstances, phosphorylation of Drp1 serine 616 (S616) enhances Drp1 activity causing more mitochondria fragmentation (53). In fMLP-stimulated neutrophils, inhibition of MCU can downregulate the phosphorylation of Drp1 S616 and impair neutrophil chemotaxis. Selective suppression of Drp1 by mdivi-1 inhibits neutrophil polarization and chemotaxis but not MCU expression and Ca^2+^ uptake, suggesting that MCU regulates neutrophil chemotaxis partially *via* Drp1 (52). More genetic evidence is needed to connect Drp1 and neutrophil polarization and chemotaxis.

Intracellular Ca^2+^ is mainly stored in the endoplasmic reticulum (ER), and the juxtaposition of ER and mitochondria is essential due to the low affinity of MCU for Ca^2+^ (51). Mitofusin2 (MFN2) stabilizes the contact between ER and mitochondria. MFN2 is involved in neutrophil migration and adhesion. Mazaki et al. found that interfering with MFN2 expression using short-hairpin RNA (shRNA) can significantly suppress the chemotaxis of neutrophil-like dHL60 cells (54). The Deng group found that MFN2-knockdown dHL60 cells have slower chemotaxis and failed to firmly adhere to endothelial cells (55), which is consistent with an *in vitro* study by Mazaki et al. They also extend their finding to an *in vivo* zebrafish model, showing that MFN2-knockout neutrophils failed to recruit to the zebrafish tail wound, and a thioglycollate-induced mouse peritonitis model that showed fewer neutrophils was recruited to the peritoneal cavity in neutrophil-specific MFN-2 knockout mice (55). Also, transfecting an artificial ER-mitochondria tether (56), mimicking MFN2, can rescue the chemotaxis defect in MFN2 knockdown dHL60 cells (55).

In collaboration with Deng’s group, our group investigated the mechanism of the adhesion defect in MFN2 knockdown dHL60 cells (57). Using microfluidic devices, we identified that MFN2 knockdown dHL60 cells have defects on β2 integrin-dependent slow rolling and arrest but not P-selectin glycoprotein ligand-1 (PSGL-1)-dependent rolling. MFN2 knockdown dHL60 cells have reduced formyl peptide receptor (FPR) expression and FPR-dependent (fMLP stimulation) and independent (PMA stimulation) β2 integrin activation defects. MFN2 knockdown dHL60 cells also show defects in actin polymerization after fMLP or PMA stimulation and Mn^2+^-induced cell spreading on ICAM-1. We demonstrated that MFN2 knockdown HL60 cells are deficient in differentiating to neutrophil-like dHL60 cells by assessing the nuclear morphology and maturation markers CD35 and CD87. Using the CD87 maturation marker, we found that in a mature CD87high population, MFN2 knockdown HL60 cells still show defects in cell slow rolling, adhesion, and β2 integrin activation, indicating that besides effects on differentiation, MFN2 is directly involved in regulating β2 integrin activation. Please note that in these mature populations, MFN2 only affects integrin extension (which is reported by the KIM127 antibody), but not headpiece opening (which is reported by the mAb24 antibody), under PMA stimulation. This suggests that MFN2 might be important for the conformational changes of bent-open to extended-open β2 integrins, which is an alternative allosteric pathway of β2 integrins we observed before (58–61), in addition to the conical switchblade model (62).

A migrasome is an extracellular organelle in migrating cells newly discovered by Yu’s group (63). During neutrophil migration, intracellular components can be released to extracellular space to form migrasomes, which is called mitocytosis. TSPAN9, a member of the tetraspanin family, is a key regulator of migrasome formation. In TSPAN9^-/-^ mice, neutrophils produced fewer migrasomes than those in WT. The number of TSPAN9^-/-^ neutrophils that can maintain mitochondria membrane potential (MMP) is reduced compared with WT, suggesting a defect in mitochondrial quality control. To directly link mitocytosis and mitochondrial quality control and exclude the other effects caused by TSPAN9 knockout, Jiao et al. compared MMP of both bone marrow and spleen neutrophils between TSPAN9-/- and WT. Since spleen neutrophils migrated a long distance while bone marrow neutrophils did not, there was less difference in MMP of bone marrow neutrophils than of spleen neutrophils, suggesting that migrasomes are mainly responsible for mitochondrial quality control (63).

## Development and differentiation

During the differentiation of neutrophils, metabolism reprogramming that limits glycolytic activity while engaging mitochondrial respiration is required. Riffelmacher et al. defined five stages of neutrophil development: myeloblasts (MBs), myelocytes (MCs), metamyelocytes (MMs), band cells (BCs), and neutrophils (PMNs) (64). Taking advantage of *Map1lc3b-*GFP transgenic mice, they found stable autophagic flux in MBs and MCs and reduced flux in MMs and BCs, which is consistent with *Tfeb* and Atg7 expression changes. Their further use of *Atg7^fl/fl^-Vav-Cre* mice revealed that knockout of Atg7 causes defects in neutrophil differentiation. Similar results were seen in *Atg5^fl/fl^-Mx1-Cre* mice, indicating that this phenomenon is not Atg7-specific. Immature neutrophils only prevalent in Atg5 -/- CD45.2^+^ but not WT CD45.1^+^ cells in bone marrow chimeric mice prove that differentiation is independent of autophagy during development. Metabolic analysis showed metabolic reprogramming during G-CSF-induced neutrophil differentiation. The increased extracellular acidification rate (ECAR) and decreased oxygen consumption rate (OCR) of Atg7^-/-^ myeloblasts suggest that metabolic reprogramming limits glycolysis and engages mitochondrial respiration. Depletion of free fatty acids (FFAs) was found in Atg7^-/-^ but not Atg7^+/+^ myeloblasts. Atg7^-/-^ neutrophil lipid droplet accumulation and colocalization of autophagosomes with lipid droplets suggest that degradation of lipid droplets is mediated by autophagy. Overall, this study showed that autophagy-mediated lipolysis provides free fatty acids to support the fatty acid β-oxidation (FAO) and OXPHOS pathway to produce ATP, which is essential for neutrophil differentiation (64).

Tanimura et al. found that both ER stress and unfolded-protein reactions (UPRs) are involved in neutrophil differentiation (65). During HL60 differentiation into neutrophils induced by ATRA, the ER-stress marker BiP expression is reduced. Treatment of 4-phenylbutyric acid (4-PBA), a chemical chaperone that can reduce ER stress, on HL60 cells can increase CD11b expression and induce morphological change. Detection of cleaved activating transcription factor (ATF) 6 and ATF4 suggests the activation of UPR in non-treated HL60 cells. Co-treatment of ATRA and UPR inhibitors on HL60 cells hindered its differentiation into neutrophils functionally and morphologically. Since both processes require ATP, they further investigated the role of mitochondrial ATP supply by using oligomycin. They found that disrupted neutrophil differentiation as a result of oligomycin treatment inhibited XBP-1 activation (65).

MFN2 conditional knockout/down mice were used to examine the role of MFN2 in neutrophil development. Luchsinger and colleagues found that in a competitive repopulation assay, *Mfn2^fl/fl^-Vav-Cre* and *Mfn2^fl/fl^* CD11b^+^Gr1^+^ myeloid cells show similar numbers in the bone marrow (66). However, they did not further study different myeloid cell populations. Zhou et al. also found that there is no significant difference in neutrophil frequencies between *Mfn2^fl/fl^-S100A8-Cre+* and *Cre*- lines. Of note, both the MFN2 transcript and protein were reduced by 50% in neutrophils of these mice (55, 57). The incomplete deletion of MFN2 suggests that the *S100A8-*Cre line may be too late for neutrophil differentiation studies. Our group used dHL60 cells to show that the MFN2 knockdown causes a neutrophil differentiation defect based on several maturation markers, including CD35, 87, 18, and nucleus segmentation (57).

## Others

Human neutrophils produced more mROS in hypoxia *ex vivo* (67). The addition of MitoTEMPO accelerates neutrophil apoptosis, suggesting that mitochondrial O_2_^–^ is needed for neutrophil survival. Neutrophils in hypoxia also display higher mitochondrial membrane potential compared to those in normoxia. Glycerol-3-phosphate dehydrogenase 2 (GPD2) is the mitochondrial component of glycerol 3-phosphate shuttle and produces mROS. To test whether GPD2 is involved in the hypoxia-induced mROS in neutrophils, Wilson et al. used iGP-1 to inhibit GPD2 and found that this inhibition led to decreased HIF-1a expression and mROS, suggesting that neutrophils use GPD2 to produce mROS and stabilize HIF-1a. Importantly, inhibition of GPD2 activity with the pro-apoptotic iGP-1 abrogates neutrophil phagocytosis of heat-killed *S. aureus* and degranulation, indicating that GPD2 activity is essential for neutrophil survival and key effector functions (67).

## Neutrophil mitochondria-associated diseases

Neutrophil dysfunctions in mitochondria have been linked to many diseases (**Table 2**). Systemic lupus erythematosus (SLE) is an autoimmune disease, and low-density granulocytes, a pro-inflammatory subset of neutrophils, from SLE patients spontaneously form more NETs. Ribonucleoprotein immune complexes (RNP-IC) are prevalent in lupus patients and can induce NETosis through an mROS-dependent manner and release oxidized mtDNA. Both *in vitro* and *ex vivo* experiments showed that oxidized mtDNA is highly inflammatory (36).

**Table 2:** Neutrophil mitochondria-associated diseases.

Diseases	Neutrophil phenotype/neutrophil deficiency	References
Systemic lupus erythematosus (SLE)	SLE low-density granulocytes spontaneously release NETs enriched in oxidized mtDNA, leading to enhanced pro-inflammatory and interferogenic potential.	(36)
ADOA	The neutrophils from ADOA patients carrying OPA1 mutations cannot release DNA following GM-CSF/C5a stimulation	(28)
Atherosclerosis	Aged mice display high mitochondrial oxidative stress and enhanced atherosclerosis development.	(68)
Rheumatoid arthritis (RA)	Synovial fluid neutrophils from rheumatoid patients display a gene expression signature of oxidative stress that leads to mtDNA release.	(69)
Adult-onset still’s disease (AOSD)	IL-18 induced mtDNA release from neutrophils, resulting in increased levels of NETs enriched in oxidized mtDNA in plasma from AOSD patients.	(37)
Breast cancer lung metastasis	Tumor-associated aged neutrophils release mitochondria-dependent NETs capturing tumor cells and promoting tumor cell retention in the lung.	(70)
Cancer immunosuppression	Tumor-elicited neutrophils maintain mitochondrial metabolism in glucose-limited TME to generate ROS and suppress T cells.	(18)

Atherosclerosis is a chronic inflammatory disorder where the roles of neutrophils in pathology are well recognized (71). NETs have been observed in atherosclerosis environments in mice and humans (72). Wang et al. used lethally irradiated low-density lipoprotein receptor-deficient mice (Ldlr^−/−^) with transplanted bone marrow from WT mice and transgenic mice containing mitochondrial catalase (mCAT), which can reduce mitochondrial oxidative stress (mitoOS) to examine the neutrophil-specific mitoOS in atherosclerosis in aged settings. As a result, aged mCAT→Ldlr^−/−^ chimeric mice had significantly less aortic root atherosclerotic lesions, fewer lesional neutrophils, and decreased NETs, compared with aged controls, suggesting the pathological role of mitoOS in atherosclerosis (68).

Rheumatoid arthritis (RA) is another chronic inflammatory disease, where osteoclasts play a vital role in the development and progression of bone loss. The receptor activator of nuclear factor kappa B ligand (RANKL) is an important factor that drives bone destruction by activating osteoclasts (73). Neutrophils highly express RANKL and activate osteoclastic bone resorption (74). Contis et al. performed a quantitative proteomic analysis of purified neutrophils isolated from synovial fluid (SF) and blood from RA patients. They found that only SF neutrophils from RA patients displayed oxidative stress gene expression signatures. They further found that SF from RA patients had a higher number of mtDNA copies than osteoarthritis patients. Moreover, the mtDNA copy number was higher in SF from RA patients with a higher disease activity score of 28, indicating that mtDNA is associated with disease severity. They also found that mtDNA can induce RANKL expression in neutrophils from healthy donors *in vitro*. Their findings suggest that neutrophils could release mtDNA in SF, which increases neutrophil RANKL expression and induce osteoclastic bone loss, forming an autocrine loop model contributing to RA progression (69).

Adult-onset Still’s disease (AOSD) is a rare type of inflammatory arthritis that features fever, rash, and joint pain. Elevated levels of NETosis are found in AOSD patients (75). Liao et al. found that MitoTEMPO but not DPI can significantly inhibit AOSD patient-serum-induced NET formation, suggesting that elevated NETs in AOSD patients are mROS-dependent. They further investigated the role of IL-18 in the NETs of AOSD and found that MitoTEMPO and calcium chelator BAPTM-AM can suppress IL-18-induced NET formation. They conclude that IL-18 can induce calcium influx into neutrophils leading to mROS production and NETosis (37).

A unique subset of neutrophils regulates the lung metastasis of breast cancer in a mitochondria-dependent way (70). Tumor-associated aged neutrophils, defined by CXCR4^+^CD62L^low^, increased in the peripheral blood and lung metastasis in the 4T1 breast tumor model and mouse mammary tumor virus-polyoma middle tumor-antigen (MMTV-PyMT) model. Further, the blood of breast cancer patients had a higher proportion of aged neutrophils compared with patients with breast fibroadenoma. Transcriptomic analysis showed that transcription factor *SIRT1* is upregulated in aged neutrophils. SIRT1 can induce NETosis, which is different compared with PMA induction in the following ways: 1) SIRT1-induced NETosis is independent of NOX and PAD4 signaling; 2) mitochondrial DNA dominates the components of SIRT1-induced neutrophil extracellular traps; and 3) SIRT1 agonist SRT1720 can activate the mitochondrial permeability transition pore. Immunofluorescence and scanning electron microscope images show that tumor-associated aged neutrophils can capture tumor cells and promote tumor cell retention in the lung through NETs. Moreover, the *SIRT1* knockdown can significantly reduce lung metastasis (70).

In cancer, neutrophils may promote tumor progression by suppressing T-cell (76–78) and NK-cell (79) activity. However, the tumor microenvironment (TME) is usually glucose-limited. As mentioned above, neutrophils mainly use glycolysis to produce energy; therefore, the glucose-limited TME may inhibit glycolysis-dependent neutrophil functions. Using 4T1 breast cancer tumor-bearing mice, Rice et al. showed that 4T1 tumors elicit the expansion of c-Kit+ neutrophils, which may indicate immaturity (18). These c-Kit+ neutrophils have increased mitochondrial metabolism that shows higher ATP synthase-dependent and maximal OCR, greater MMP, and higher expression of complexes I and IV. Moreover, these c-Kit+ neutrophils use mitochondrial fatty acid oxidation to support stronger NADPH oxidase-dependent ROS production while limiting glucose compared to neutrophils from naïve mice. The authors further hypothesize that tumor-produced stem cell factor (SCF), a c-Kit ligand, maintains mitochondrial metabolism *via* the c-Kit–SCF axis. Indeed, after using c-Kit antibody blockade or SCF-null tumors, neutrophil number and mitochondrial activity are decreased. They conclude that tumor-elicited c-Kit signaling is responsible for the increased neutrophil number and neutrophil oxidative adaptation. In the coculture assay, tumor-elicited neutrophils stimulated with PMA can induce significantly more T-cell death and suppress T-cell proliferation and interferon-γ (IFN-γ) production compared to neutrophils from naïve mice. Further, suppressing neutrophil glycolysis by 2-deoxy-D-glucose pretreatment did not abolish the T-cell killing ability of tumor-elicited neutrophils. Consistent with their mouse data, they found that neutrophils from peripheral blood of ovarian cancer patients have higher ATP synthase-dependent OCR.

## Discussion

Neutrophil mitochondria participate in many neutrophil functions, such as respiratory burst, NET formation, migration, adhesion, differentiation/development, and degranulation. Dysfunction of neutrophil mitochondria is associated with many human diseases, such as cancer, lupus, and rheumatoid arthritis. In-depth investigations of mitochondria help people realize the heterogeneous and complicated nature of the neutrophil population, providing new insights into neutrophil biology and uncovering new targets to treat diseases.

Current research studying mitochondria and their components rely heavily on pharmacological inhibitors. Thus, the off-target effects of these inhibitors may lead to misleading interpretations. Gene editing of mitochondria-specific genes (33, 43, 55, 57) provides more precise interpretations of the function of different mitochondrial components in regulating neutrophil functions. However, the global knockout of most mitochondrial genes will cause severe developmental defects. Thus, several investigations were performed in the neutrophil-like HL60 cell line. Whether these findings are consistent in primary neutrophils remains to be further investigated. The conditional knockout using the Cre-loxP system provides a tool to study mitochondrial components in mouse neutrophils. Some cres, such as MRP8-cre (S100a8-cre) (80, p.; 55, 81) and Ly6G-cre (82, 83), are specific for neutrophils. The LysM-cre (80, 84, 85) targets both neutrophils and monocytes/macrophages. Since neutrophils are short-lived cells, even the genes of mitochondrial components are knocked out, and some cres are not efficient in eliminating protein expression of mitochondrial components. For example, in our study using MFN2^flox/flox^MRP8^cre^ mice (57), we found that MFN2 protein expression is only decreased by 50-60% in peripheral blood neutrophils. A cre expressing in an earlier stage of hematopoietic differentiation, such as Vav1-cre (80, 86), might increase the knockout efficiency but will lose the specificity to neutrophils.

An alternative to studying the roles of mitochondrial components in neutrophils is to use HoxB8-expressing neutrophil progenitors (87). HoxB8 expression blocks the terminal differentiation of progenitors into monocytes or granulocytes (88, 89). Gene editing can be done in HoxB8 neutrophil progenitors. HoxB8 neutrophil progenitors can differentiate into neutrophils *in vitro* (90–94) and *in vivo* (95, 96). These will provide a powerful tool to study the involvement of mitochondrial components in mouse neutrophils.

There are strategies to perform gene editing in human neutrophils and study the roles of mitochondrial components. One is using a highly effective di-peptide caspase inhibitor, Q-VD.OPh, to inhibit apoptosis in long-time *in vitro* culture of human neutrophils (97). However, whether it is possible to perform gene editing of human neutrophils in this system and whether this culture will affect some neutrophil functions is not clear. A better strategy is to use human-induced pluripotent stem cells (iPSCs), perform gene editing on them, and differentiate them into neutrophils (98–101). This may be the best strategy to study the roles of mitochondrial components in human neutrophils.

Besides nuclear genome-encoded genes of mitochondrial components, mitochondrial DNA also encodes genes that are associated with many diseases, such as Leigh syndrome (102–104), NARP (neuron, ataxia, and retinitis pigmentosa) syndrome (105), and Leber hereditary optic neuropathy (106, 107). Thus, mitochondrial gene editing is critical for studying the roles of mitochondria in human neutrophils. Several techniques to edit mitochondrial genes were highlighted in previous reviews (108, 109). One of the techniques used a modulated Cas9-CRISPR gene-editing technique by appending a gene targeting guide RNA to an RNA transport-derived stem loop element (RP-loop) and expressing the Cas9 enzyme with a preceding mitochondrial localization sequence (110). Another technique is using an interbacterial toxin, DddA, which catalyzes cytidine deamination within double-stranded DNA. Fusions of the split-DddA halves, transcription activator-like effector array proteins, and a uracil glycosylase inhibitor resulted in RNA-free DddA-derived cytosine base editors (DdCBEs) that catalyze C·G-to-T·A conversions in human mtDNA with high targeting specificity and product purity (111). Later, another technique using transcription-activator-like effector (TALE)-linked deaminases (TALEDs) can catalyze A·T-to-G·C conversions in human mtDNA (112). These mitochondrial gene-editing techniques will bring new insights into studying the roles of mitochondria in neutrophil functions.

## Author contributions

ZC drafted the manuscript; ZC prepared the figure and tables; HS, LH, YC, MZ, and ZF edited and revised the manuscript; ZF approved the final version of the manuscript. All authors contributed to the article and approved the submitted version.

## Funding

This research was supported by funding from the National Institutes of Health, USA (NIH, R01HL145454), and a startup fund from UConn Health.

## Acknowledgments

We acknowledge Dr. Christopher “Kit” Bonin and Dr. Geneva Hargis from UConn Health School of Medicine for their help scientific writing and editing of this manuscript.

## Conflict of interest

The authors declare that the research was conducted in the absence of any commercial or financial relationships that could be construed as a potential conflict of interest.

## Publisher’s note

All claims expressed in this article are solely those of the authors and do not necessarily represent those of their affiliated organizations, or those of the publisher, the editors and the reviewers. Any product that may be evaluated in this article, or claim that may be made by its manufacturer, is not guaranteed or endorsed by the publisher.

## References

[1] Kolaczkowska E Kubes P. Neutrophil recruitment and function in health and inflammation. Nat Rev Immunol (2013) 13(3):159–75. 10.1038/nri3399 23435331

[2] Ley K Laudanna C Cybulsky MI Nourshargh S. Getting to the site of inflammation: The leukocyte adhesion cascade updated. Nat Rev Immunol (2007) 7(9):678–89. 10.1038/nri2156 17717539

[3] Ley K Hoffman HM Kubes P Cassatella MA Zychlinsky A Hedrick CC. Neutrophils: New insights and open questions. Sci Immunol (2018) 3(30):eaat4579. 30530726 10.1126/sciimmunol.aat4579

[4] Morikis VA Simon SI. Neutrophil mechanosignaling promotes integrin engagement with endothelial cells and motility within inflamed vessels. Front Immunol (2018) 2774. 10.3389/fimmu.2018.02774 PMC6279920 30546362

[5] Boero E Brinkman I Juliet T van Yperen E van Strijp JAG Rooijakkers SHM. Use of flow cytometry to evaluate phagocytosis of staphylococcus aureus by human neutrophils. Front Immunol (2021) 12:635825. doi: 10.3389/fimmu.2021.635825 33679791 PMC7934835

[6] Gierlikowska B Stachura A Gierlikowski W Demkow U. Phagocytosis, degranulation and extracellular traps release by neutrophils–the current knowledge, pharmacological modulation and future prospects. Front Pharmacol (2021) 12:666732. doi: 10.3389/fphar.2021.666732 34017259 PMC8129565

[7] Payne JA Tailhades J Ellett F Kostoulias X Fulcher AJ Fu T. Antibiotic-chemoattractants enhance neutrophil clearance of staphylococcus aureus. Nat Commun (2021) 12(1):1–15. 34697316 10.1038/s41467-021-26244-5 PMC8546149

[8] Brinkmann V Reichard U Goosmann C Fauler B Uhlemann Y Weiss DS. Neutrophil extracellular traps kill bacteria. Science (2004) 303(5663):1532–5. 10.1126/science.1092385 15001782

[9] Monteith AJ Miller JM Maxwell CN Chazin WJ Skaar EP. Neutrophil extracellular traps enhance macrophage killing of bacterial pathogens. Sci Adv (2021) 7(37):eabj2101. 34516771 10.1126/sciadv.abj2101 PMC8442908

[10] Eichelberger KR Goldman WE. Manipulating neutrophil degranulation as a bacterial virulence strategy. PLoS Pathog (2020) 16(12):e1009054. doi: 10.1371/journal.ppat.1009054 33301542 PMC7728292

[11] Beavers WN Skaar EP. Neutrophil-generated oxidative stress and protein damage in staphylococcus aureus. Pathog Dis (2016) 74(6):ftw060. doi: 10.1093/femspd/ftw060 27354296 PMC5975594

[12] Nauseef WM. How human neutrophils kill and degrade microbes: An integrated view. Immunol Rev (2007) 219(1):88–102. 17850484 10.1111/j.1600-065X.2007.00550.x

[13] Bock FJ Tait SW. Mitochondria as multifaceted regulators of cell death. Nat Rev Mol Cell Biol (2020) 21(2):85–100. 31636403 10.1038/s41580-019-0173-8

[14] Fossati G Moulding DA Spiller DG Moots RJ White MR Edwards SW. The mitochondrial network of human neutrophils: Role in chemotaxis, phagocytosis, respiratory burst activation, and commitment to apoptosis. J Immunol (2003) 170(4):1964–72. 10.4049/jimmunol.170.4.1964 12574365

[15] Bao Y Ledderose C Seier T Graf AF Brix B Chong E. Mitochondria regulate neutrophil activation by generating ATP for autocrine purinergic signaling. J Biol Chem (2014) 289(39):26794–803. 10.1074/jbc.M114.572495 PMC4175322 25104353

[16] Vorobjeva N Prikhodko A Galkin I Pletjushkina O Zinovkin R Sud’ina G. Mitochondrial reactive oxygen species are involved in chemoattractant-induced oxidative burst and degranulation of human neutrophils *in vitro* . Eur J Cell Biol (2017) 96(3):254–65. 10.1016/j.ejcb.2017.03.003 28325500

[17] Dunham-Snary KJ Surewaard BG Mewburn JD Bentley RE Martin AY Jones O. Mitochondria in human neutrophils mediate killing of staphylococcus aureus. Redox Biol (2022) 49:102225. 34959099 10.1016/j.redox.2021.102225 PMC8758915

[18] Rice CM Davies LC Subleski JJ Maio N Gonzalez-Cotto M Andrews C. Tumour-elicited neutrophils engage mitochondrial metabolism to circumvent nutrient limitations and maintain immune suppression. Nat Commun (2018) 9(1):1–13. 30504842 10.1038/s41467-018-07505-2 PMC6269473

[19] Fuchs TA Abed U Goosmann C Hurwitz R Schulze I Wahn V. Novel cell death program leads to neutrophil extracellular traps. J Cell Biol (2007) 176(2):231–41. 10.1083/jcb.200606027 PMC2063942 17210947

[20] Castanheira FV Kubes P. Neutrophils and NETs in modulating acute and chronic inflammation. Blood J Am Soc Hematol (2019) 133(20):2178–85. 10.1182/blood-2018-11-844530 30898862

[21] Papayannopoulos V. Neutrophil extracellular traps in immunity and disease. Nat Rev Immunol (2018) 18(2):134–47. 10.1038/nri.2017.105 28990587

[22] Hosseinzadeh A Thompson PR Segal BH Urban CF. Nicotine induces neutrophil extracellular traps. J Leukoc Biol (2016) 100(5):1105–12. 10.1189/jlb.3AB0815-379RR PMC5069087 27312847

[23] Hakkim A Fuchs TA Martinez NE Hess S Prinz H Zychlinsky A. Activation of the raf-MEK-ERK pathway is required for neutrophil extracellular trap formation. Nat Chem Biol (2011) 7(2):75–7. 10.1038/nchembio.496 21170021

[24] Papayannopoulos V Metzler KD Hakkim A Zychlinsky A. Neutrophil elastase and myeloperoxidase regulate the formation of neutrophil extracellular traps. J Cell Biol (2010) 191(3):677–91. 10.1083/jcb.201006052 PMC3003309 20974816

[25] Azevedo EP Rochael NC Guimarães-Costa AB de Souza-Vieira TS Ganilho J Saraiva EM. A metabolic shift toward pentose phosphate pathway is necessary for amyloid fibril-and phorbol 12-myristate 13-acetate-induced neutrophil extracellular trap (NET) formation. J Biol Chem (2015) 290(36):22174–83. 10.1074/jbc.M115.640094 PMC4571968 26198639

[26] Rodríguez-Espinosa O Rojas-Espinosa O Moreno-Altamirano MMB López-Villegas EO Sánchez-García FJ. Metabolic requirements for neutrophil extracellular traps formation. Immunology (2015) 145(2):213–24. 10.1111/imm.12437 PMC4427386 25545227

[27] Yousefi S Mihalache C Kozlowski E Schmid I Simon H-U. Viable neutrophils release mitochondrial DNA to form neutrophil extracellular traps. Cell Death Differ (2009) 16(11):1438–44. 10.1038/cdd.2009.96 19609275

[28] Amini P Stojkov D Felser A Jackson CB Courage C Schaller A. Neutrophil extracellular trap formation requires OPA1-dependent glycolytic ATP production. Nat Commun (2018) 9(1):1–16. 30054480 10.1038/s41467-018-05387-y PMC6063938

[29] Douda DN Khan MA Grasemann H Palaniyar N. SK3 channel and mitochondrial ROS mediate NADPH oxidase-independent NETosis induced by calcium influx. Proc Natl Acad Sci (2015) 112(9):2817–22. 10.1073/pnas.1414055112 PMC4352781 25730848

[30] Takishita Y Yasuda H Shimizu M Matsuo A Morita A Tsutsumi T. Formation of neutrophil extracellular traps in mitochondrial DNA-deficient cells. J Clin Biochem Nutr (2019) 66(1):15–23. 32001952 10.3164/jcbn.19-77 PMC6983440

[31] Naffah de Souza C Breda LCD Khan MA Almeida SR Câmara NOS Sweezey N. Alkaline pH promotes NADPH oxidase-independent neutrophil extracellular trap formation: A matter of mitochondrial reactive oxygen species generation and citrullination and cleavage of histone. Front Immunol (2018) 8:1849. doi: 10.3389/fimmu.2017.01849 29375550 PMC5767187

[32] Reithofer M Karacs J Strobl J Kitzmüller C Polak D Seif K. Alum triggers infiltration of human neutrophils ex vivo and causes lysosomal destabilization and mitochondrial membrane potential-dependent NET-formation. FASEB J (2020) 34(10):14024–41. 10.1096/fj.202001413R PMC7589265 32860638

[33] Monteith AJ Miller JM Beavers WN Maloney KN Seifert EL Hajnoczky G. Mitochondrial calcium uniporter affects neutrophil bactericidal activity during staphylococcus aureus infection. Infect Immun (2021) 90(2):e0055121. 34871043 10.1128/iai.00551-21 PMC8853686

[34] Lewis HD Liddle J Coote JE Atkinson SJ Barker MD Bax BD. Inhibition of PAD4 activity is sufficient to disrupt mouse and human NET formation. Nat Chem Biol (2015) 11(3):189–91. 10.1038/nchembio.1735 PMC4397581 25622091

[35] Li P Li M Lindberg MR Kennett MJ Xiong N Wang Y. PAD4 is essential for antibacterial innate immunity mediated by neutrophil extracellular traps. J Exp Med (2010) 207(9):1853–62. doi: 10.1084/jem.20100239 PMC2931169 20733033

[36] Lood C Blanco LP Purmalek MM Carmona-Rivera C De Ravin SS Smith CK. Neutrophil extracellular traps enriched in oxidized mitochondrial DNA are interferogenic and contribute to lupus-like disease. Nat Med (2016) 22(2):146–53. 10.1038/nm.4027 PMC4742415 26779811

[37] Liao T-L Chen Y-M Tang K-T Chen P-K Liu H-J Chen D-Y. MicroRNA-223 inhibits neutrophil extracellular traps formation through regulating calcium influx and small extracellular vesicles transmission. Sci Rep (2021) 11(1):1–17. 34344968 10.1038/s41598-021-95028-0 PMC8333426

[38] Maianski N Geissler J Srinivasula S Alnemri E Roos D Kuijpers T. Functional characterization of mitochondria in neutrophils: A role restricted to apoptosis. Cell Death Differ (2004) 11(2):143–53. 10.1038/sj.cdd.4401320 14576767

[39] Bao Y Ledderose C Graf AF Brix B Birsak T Lee A. MTOR and differential activation of mitochondria orchestrate neutrophil chemotaxis. J Cell Biol (2015) 210(7):1153–64. 10.1083/jcb.201503066 PMC4586745 26416965

[40] Chen Y Corriden R Inoue Y Yip L Hashiguchi N Zinkernagel A. ATP release guides neutrophil chemotaxis *via* P2Y2 and A3 receptors. Science (2006) 314(5806):1792–5. 10.1126/science.1132559 17170310

[41] Kondo Y Ledderose C Slubowski CJ Fakhari M Sumi Y Sueyoshi K. Frontline science: Escherichia coli use LPS as decoy to impair neutrophil chemotaxis and defeat antimicrobial host defense. J Leukoc Biol (2019) 106(6):1211–9. doi: 10.1002/JLB.4HI0319-109R PMC6883117 31392789

[42] Tavares-Murta BM Zaparoli M Ferreira RB Silva-Vergara ML Oliveira CH Murta EFC. Failure of neutrophil chemotactic function in septic patients*. Crit Care Med (2002) 30(5):1056–61. 10.1097/00003246-200205000-00017 12006803

[43] Zhou W Cao L Jeffries J Zhu X Staiger CJ Deng Q. Neutrophil-specific knockout demonstrates a role for mitochondria in regulating neutrophil motility in zebrafish. Dis Models Mech (2018) 11(3):dmm033027. 10.1242/dmm.033027 PMC5897731 29590639

[44] Sakai J Li J Subramanian KK Mondal S Bajrami B Hattori H. Reactive oxygen species-induced actin glutathionylation controls actin dynamics in neutrophils. Immunity (2012) 37(6):1037–49. 10.1016/j.immuni.2012.08.017 PMC3525814 23159440

[45] Zmijewski JW Lorne E Zhao X Tsuruta Y Sha Y Liu G. Mitochondrial respiratory complex I regulates neutrophil activation and severity of lung injury. Am J Respir Crit Care Med (2008) 178(2):168–79. 10.1164/rccm.200710-1602OC PMC2453511 18436790

[46] Mimaki M Wang X McKenzie M Thorburn DR Ryan MT. Understanding mitochondrial complex I assembly in health and disease. Biochim Biophys Acta (2012) 1817:851–62. doi: 10.1016/j.bbabio.2011.08.010 21924235

[47] Smith PM Fox JL Winge DR. Biogenesis of the cytochrome bc(1) complex and role of assembly factors. Biochim Biophys Acta (2012) 1817:276–86. doi: 10.1016/j.bbabio.2011.11.009 PMC3366459 22138626

[48] El Chemaly A Okochi Y Sasaki M Arnaudeau S Okamura Y Demaurex N. VSOP/Hv1 proton channels sustain calcium entry, neutrophil migration, and superoxide production by limiting cell depolarization and acidification. J Exp Med (2010) 207(1):129–39. 10.1084/jem.20091837 PMC2812533 20026664

[49] Okochi Y Umemoto E Okamura Y. Hv1/VSOP regulates neutrophil directional migration and ERK activity by tuning ROS production. J Leukoc Biol (2020) 107(5):819–31. 10.1002/JLB.2A0320-110RR 32303121

[50] Riganti C Gazzano E Polimeni M Costamagna C Bosia A Ghigo D. Diphenyleneiodonium inhibits the cell redox metabolism and induces oxidative stress. J Biol Chem (2004) 279(46):47726–31. 10.1074/jbc.M406314200 15358777

[51] Paupe V Prudent J. New insights into the role of mitochondrial calcium homeostasis in cell migration. Biochem Biophys Res Commun (2018) 500(1):75–86. 28495532 10.1016/j.bbrc.2017.05.039 PMC5930976

[52] Zheng X Chen M Meng X Chu X Cai C Zou F. Phosphorylation of dynamin-related protein 1 at Ser616 regulates mitochondrial fission and is involved in mitochondrial calcium uniporter-mediated neutrophil polarization and chemotaxis. Mol Immunol (2017) 87:23–32. 28388446 10.1016/j.molimm.2017.03.019

[53] Cho B Choi SY Cho HM Kim HJ Sun W. Physiological and pathological significance of dynamin-related protein 1 (drp1)-dependent mitochondrial fission in the nervous system. Exp Neurobiol (2013) 22(3):149. 24167410 10.5607/en.2013.22.3.149 PMC3807002

[54] Mazaki Y Takada S Nio-Kobayashi J Maekawa S Higashi T Onodera Y. Mitofusin 2 is involved in chemotaxis of neutrophil-like differentiated HL-60 cells. Biochem Biophys Res Commun (2019) 513(3):708–13. 10.1016/j.bbrc.2019.04.037 30987827

[55] Zhou W Hsu AY Wang Y Syahirah R Wang T Jeffries J. Mitofusin 2 regulates neutrophil adhesive migration and the actin cytoskeleton. J Cell Sci (2020) 133(17):jcs248880. 32788232 10.1242/jcs.248880 PMC7491649

[56] Kornmann B Currie E Collins SR Schuldiner M Nunnari J Weissman JS. An ER-mitochondria tethering complex revealed by a synthetic biology screen. Science (2009) 325(5939):477–81. 10.1126/science.1175088 PMC2933203 19556461

[57] Liu W Hsu AY Wang Y Lin T Sun H Pachter JS. Mitofusin-2 regulates leukocyte adhesion and β2 integrin activation. J Leukoc Biol (2021) 111(4):771–91. 10.1002/JLB.1A0720-471R PMC8901796 34494308

[58] Fan Z McArdle S Marki A Mikulski Z Gutierrez E Engelhardt B. Neutrophil recruitment limited by high-affinity bent β2 integrin binding ligand in cis. Nat Commun (2016) 7(1):1–14. 10.1038/ncomms12658 PMC5013657 27578049

[59] Fan Z Kiosses WB Sun H Orecchioni M Ghosheh Y Zajonc DM. High-affinity bent β2-integrin molecules in arresting neutrophils face each other through binding to ICAMs in cis. Cell Rep (2019) 26(1):119–30. 10.1016/j.celrep.2018.12.038 PMC6625519 30605669

[60] Sun H Hu L Fan Z. β2 integrin activation and signal transduction in leukocyte recruitment. Am J Physiol-Cell. Physiol (2021) 321(2):C308–16. 10.1152/ajpcell.00560.2020 PMC8424673 34133240

[61] Sun H Zhi K Hu L Fan Z. The activation and regulation of β2 integrins in phagocytes. Front Immunol (2021) 12:978. 10.3389/fimmu.2021.633639 PMC8044391 33868253

[62] Luo B-H Carman CV Springer TA. Structural basis of integrin regulation and signaling. Annu Rev Immunol (2007) 25:619–47. 10.1146/annurev.immunol.25.022106.141618 PMC1952532 17201681

[63] Jiao H Jiang D Hu X Du W Ji L Yang Y. Mitocytosis, a migrasome-mediated mitochondrial quality-control process. Cell (2021) 184(11):2896–910. 10.1016/j.cell.2021.04.027 34048705

[64] Riffelmacher T Clarke A Richter FC Stranks A Pandey S Danielli S. Autophagy-dependent generation of free fatty acids is critical for normal neutrophil differentiation. Immunity (2017) 47(3):466–80. 10.1016/j.immuni.2017.08.005 PMC5610174 28916263

[65] Tanimura A Miyoshi K Horiguchi T Hagita H Fujisawa K Noma T. Mitochondrial activity and unfolded protein response are required for neutrophil differentiation. Cell Physiol Biochem (2018) 47(5):1936–50. 10.1159/000491464 29972819

[66] Luchsinger LL de Almeida MJ Corrigan DJ Mumau M Snoeck H-W. Mitofusin 2 maintains haematopoietic stem cells with extensive lymphoid potential. Nature (2016) 529(7587):528–31. 10.1038/nature16500 PMC5106870 26789249

[67] Willson JA Arienti S Sadiku P Reyes L Coelho P Morrison T. Neutrophil HIF-1α stabilization is augmented by mitochondrial ROS produced *via* the glycerol 3-phosphate shuttle. Blood J Am Soc Hematol (2022) 139(2):281–6. 10.1182/blood.2021011010 PMC8832465 34411229

[68] Wang Y Wang W Wang N Tall AR Tabas I. Mitochondrial oxidative stress promotes atherosclerosis and neutrophil extracellular traps in aged mice. Arteriosclerosis. Thromb. Vasc Biol (2017) 37(8):e99–e107. 10.1161/ATVBAHA.117.309580 PMC5535797 28596373

[69] Contis A Mitrovic S Lavie J Douchet I Lazaro E Truchetet M-E. Neutrophil-derived mitochondrial DNA promotes receptor activator of nuclear factor κB and its ligand signalling in rheumatoid arthritis. Rheumatology (2017) 56(7):1200–5. 10.1093/rheumatology/kex041 28340056

[70] Yang C Wang Z Li L Zhang Z Jin X Wu P. Aged neutrophils form mitochondria-dependent vital NETs to promote breast cancer lung metastasis. J Immunotherapy. Cancer (2021) 9(10):e002875. 10.1136/jitc-2021-002875 PMC8559246 34716206

[71] Soehnlein O. Multiple roles for neutrophils in atherosclerosis. Circ Res (2012) 110(6):875–88. 10.1161/CIRCRESAHA.111.257535 22427325

[72] Megens RT Vijayan S Lievens D Doering Y van Zandvoort MA Grommes J. Presence of luminal neutrophil extracellular traps in atherosclerosis. Thromb Haemostasis (2012) 107(03):597–8. 10.1160/TH11-09-0650 22318427

[73] Yeo L Toellner K-M Salmon M Filer A Buckley CD Raza K. Cytokine mRNA profiling identifies b cells as a major source of RANKL in rheumatoid arthritis. Ann Rheumatic. Dis (2011) 70(11):2022–8. 10.1136/ard.2011.153312 PMC3184241 21742639

[74] Chakravarti A Raquil M-A Tessier P Poubelle PE. Surface RANKL of toll-like receptor 4–stimulated human neutrophils activates osteoclastic bone resorption. Blood J Am Soc Hematol (2009) 114(8):1633–44. 10.1182/blood-2008-09-178301 19546479

[75] Hu Q Shi H Zeng T Liu H Su Y Cheng X. Increased neutrophil extracellular traps activate NLRP3 and inflammatory macrophages in adult-onset Still's disease. Arthritis Res Ther (2019) 21(1):276–86. doi: 10.1186/s13075-018-1800-z PMC6323819 30616678

[76] Kalyan S Kabelitz D. When neutrophils meet T cells: Beginnings of a tumultuous relationship with underappreciated potential. Eur J Immunol (2014) 44(3):627–33. 10.1002/eji.201344195 24435886

[77] Leliefeld PH Koenderman L Pillay J. How neutrophils shape adaptive immune responses. Front Immunol (2015) 6:471. 26441976 10.3389/fimmu.2015.00471 PMC4568410

[78] Pillay J Tak T Kamp VM Koenderman L. Immune suppression by neutrophils and granulocytic myeloid-derived suppressor cells: Similarities and differences. Cell Mol Life Sci (2013) 70(20):3813–27. 10.1007/s00018-013-1286-4 PMC3781313 23423530

[79] Spiegel A Brooks MW Houshyar S Reinhardt F Ardolino M Fessler E. Neutrophils suppress intraluminal NK cell–mediated tumor cell clearance and enhance extravasation of disseminated carcinoma CellsNeutrophil-mediated tumor cell survival and extravasation. Cancer Discovery (2016) 6(6):630–49. 10.1158/2159-8290.CD-15-1157 PMC4918202 27072748

[80] Abram CL Roberge GL Hu Y Lowell CA. Comparative analysis of the efficiency and specificity of myeloid-cre deleting strains using ROSA-EYFP reporter mice. J Immunol Methods (2014) 408:89–100. 24857755 10.1016/j.jim.2014.05.009 PMC4105345

[81] Passegué E Wagner EF Weissman IL. JunB deficiency leads to a myeloproliferative disorder arising from hematopoietic stem cells. Cell (2004) 119(3):431–43. 10.1016/j.cell.2004.10.010 15507213

[82] Ancey P-B Contat C Boivin G Sabatino S Pascual J Zangger N. GLUT1 expression in tumor-associated neutrophils promotes lung cancer growth and resistance to radiotherapy. Cancer Res (2021) 81(9):2345–57. 10.1158/0008-5472.CAN-20-2870 PMC8137580 33753374

[83] Hasenberg A Hasenberg M Männ L Neumann F Borkenstein L Stecher M. Catchup: A mouse model for imaging-based tracking and modulation of neutrophil granulocytes. Nat Methods (2015) 12(5):445–52. 10.1038/nmeth.3322 25775045

[84] Clausen B Burkhardt C Reith W Renkawitz R Förster I. Conditional gene targeting in macrophages and granulocytes using LysMcre mice. Transgenic Res (1999) 8(4):265–77. 10.1023/a:1008942828960 10621974

[85] Shi J Hua L Harmer D Li P Ren G. Cre driver mice targeting macrophages. In: Methods Mol Biol. Springer (2018). 1784:263–75. 10.1007/978-1-4939-7837-3_24 PMC6331202 29761406

[86] de Boer J Williams A Skavdis G Harker N Coles M Tolaini M. Transgenic mice with hematopoietic and lymphoid specific expression of cre. Eur J Immunol (2003) 33(2):314–25. 10.1002/immu.200310005 12548562

[87] Wang GG Calvo KR Pasillas MP Sykes DB Häcker H Kamps MP. Quantitative production of macrophages or neutrophils ex vivo using conditional Hoxb8. Nat Methods (2006) 3(4):287–93. 10.1038/nmeth865 16554834

[88] Knoepfler PS Sykes DB Pasillas M Kamps MP. HoxB8 requires its pbx-interaction motif to block differentiation of primary myeloid progenitors and of most cell line models of myeloid differentiation. Oncogene (2001) 20(39):5440–8. 10.1038/sj.onc.1204710 11571641

[89] Krishnaraju K Hoffman B Liebermann DA. Lineage-specific regulation of hematopoiesis by HOX-B8 (HOX-2.4): Inhibition of granulocytic differentiation and potentiation of monocytic differentiation. Blood J Am Soc Hematol (1997) 90(5):1840–9. 9292516

[90] Chu JY McCormick B Mazelyte G Michael M Vermeren S. HoxB8 neutrophils replicate fcγ receptor and integrin-induced neutrophil signaling and functions. J Leukoc. Biol (2019) 105(1):93–100. 30211955 10.1002/JLB.1AB0618-232R PMC6348421

[91] McDonald JU Cortini A Rosas M Fossati-Jimack L Ling GS Lewis KJ. *In vivo* functional analysis and genetic modification of *in vitro*-derived mouse neutrophils. FASEB J (2011) 25(6):1972–82. 10.1096/fj.10-178517 21368104

[92] Saul S Castelbou C Fickentscher C Demaurex N. Signaling and functional competency of neutrophils derived from bone-marrow cells expressing the ER-HOXB8 oncoprotein. J Leukoc Biol (2019) 106(5):1101–15. 10.1002/JLB.2A0818-314R 31216372

[93] Weiss E Hanzelmann D Fehlhaber B Klos A von Loewenich FD Liese J. Formyl-peptide receptor 2 governs leukocyte influx in local staphylococcus aureus infections. FASEB J (2018) 32(1):26–36. 28855276 10.1096/fj.201700441R PMC5731131

[94] Zehrer A Pick R Salvermoser M Boda A Miller M Stark K. A fundamental role of Myh9 for neutrophil migration in innate immunity. J Immunol (2018) 201(6):1748–64. 10.4049/jimmunol.1701400 30068598

[95] Cohen JT Danise M Hinman KD Neumann BM Johnson R Wilson ZS. Engraftment, fate, and function of HoxB8-conditional neutrophil progenitors in the unconditioned murine host. Front Cell Dev Biol (2022) 41. 10.3389/fcell.2022.840894 PMC8812959 35127689

[96] Orosz A Walzog B Mócsai A. *In vivo* functions of mouse neutrophils derived from hoxb8-transduced conditionally immortalized myeloid progenitors. J Immunol (2021) 206(2):432–45. 10.4049/jimmunol.2000807 33310871

[97] Wardle DJ Burgon J Sabroe I Bingle CD Whyte MK Renshaw SA. Effective caspase inhibition blocks neutrophil apoptosis and reveals mcl-1 as both a regulator and a target of neutrophil caspase activation. PLoS One (2011) 6(1):e15768. 21253591 10.1371/journal.pone.0015768 PMC3017075

[98] Brok-Volchanskaya VS Bennin DA Suknuntha K Klemm LC Huttenlocher A Slukvin I. Effective and rapid generation of functional neutrophils from induced pluripotent stem cells using ETV2-modified mRNA. Stem Cell Rep (2019) 13(6):1099–110. 10.1016/j.stemcr.2019.10.007 PMC6915846 31708474

[99] Majumder A Suknuntha K Bennin D Klemm L Brok-Volchanskaya VS Huttenlocher A. Generation of human neutrophils from induced pluripotent stem cells in chemically defined conditions using ETV2 modified mRNA. STAR. Protoc (2020) 1(2):100075. 33043305 10.1016/j.xpro.2020.100075 PMC7543976

[100] Trump LR Nayak RC Singh AK Emberesh S Wellendorf AM Lutzko CM. Neutrophils derived from genetically modified human induced pluripotent stem cells circulate and phagocytose bacteria *in vivo* . Stem Cells Trans Med (2019) 8(6):557–67. 10.1002/sctm.18-0255 PMC6525559 30793529

[101] Tsui M Min W Ng S Dobbs K Notarangelo LD Dror Y. The use of induced pluripotent stem cells to study the effects of adenosine deaminase deficiency on human neutrophil development. Front Immunol (2021) 12. 10.3389/fimmu.2021.748519 PMC8582638 34777360

[102] Pelnena D Burnyte B Jankevics E Lace B Dagyte E Grigalioniene K. Complete mtDNA sequencing reveals mutations m. 9185T> c and m. 13513G> a in three patients with Leigh syndrome. Mitochondrial. DNA Part A (2018) 29(7):1115–20. 10.1080/24701394.2017.1413365 29228836

[103] Sofou K de Coo IF Ostergaard E Isohanni P Naess K De Meirleir L. Phenotype-genotype correlations in Leigh syndrome: New insights from a multicentre study of 96 patients. J Med Genet (2018) 55(1):21–7. 10.1136/jmedgenet-2017-104891 29101127

[104] Wei Y Cui L Peng B. Mitochondrial DNA mutations in late-onset Leigh syndrome. J Neurol (2018) 265(10):2388–95. 10.1007/s00415-018-9014-5 30128709

[105] Mordel P Schaeffer S Dupas Q Laville M-A Gérard M Chapon F. A 2 bp deletion in the mitochondrial ATP 6 gene responsible for the NARP (neuropathy, ataxia, and retinitis pigmentosa) syndrome. Biochem Biophys Res Commun (2017) 494(1–2):133–7. 10.1016/j.bbrc.2017.10.066 29054413

[106] Carelli V Carbonelli M Irenaeus F Kawasaki A Klopstock T Lagrèze WA. International consensus statement on the clinical and therapeutic management of leber hereditary optic neuropathy. J Neuro-Ophthalmology (2017) 37(4):371–81. 10.1097/WNO.0000000000000570 28991104

[107] Wallace DC Singh G Lott MT Hodge JA Schurr TG Lezza AM. Mitochondrial DNA mutation associated with leber’s hereditary optic neuropathy. Science (1988) 242(4884):1427–30. 10.1126/science.3201231 3201231

[108] Yang X Jiang J Li Z Liang J Xiang Y. Strategies for mitochondrial gene editing. Comput Struct Biotechnol J (2021) 19:3319–29. 10.1016/j.csbj.2021.06.003 PMC8202187 34188780

[109] Yin T Luo J Huang D Li H. Current progress of mitochondrial genome editing by CRISPR. Front Physiol (2022) 884. 10.3389/fphys.2022.883459 PMC9108280 35586709

[110] Hussain S-RA Yalvac ME Khoo B Eckardt S McLaughlin KJ. Adapting CRISPR/Cas9 system for targeting mitochondrial genome. Front Genet (2021) 12:627050. 33889176 10.3389/fgene.2021.627050 PMC8055930

[111] Mok BY de Moraes MH Zeng J Bosch DE Kotrys AV Raguram A. A bacterial cytidine deaminase toxin enables CRISPR-free mitochondrial base editing. Nature (2020) 583(7817):631–7. 10.1038/s41586-020-2477-4 PMC7381381 32641830

[112] Cho S-I Lee S Mok YG Lim K Lee J Lee JM. Targeted a-to-G base editing in human mitochondrial DNA with programmable deaminases. Cell (2022) 185(10):1764–76. 10.1016/j.cell.2022.03.039 35472302

